# Effect of additives and moisture on the fermentation quality and bacterial community of high moisture ear corn

**DOI:** 10.3389/fmicb.2023.1251946

**Published:** 2023-11-29

**Authors:** Jiajun Li, Zheng Li, Songlin Shang, Xi Zhao, Wenjing Zhang, Xinrui Zhang, Jinni Bai, Zhiye Yang, Kaijun Guo

**Affiliations:** ^1^College of Animal Science and Technology, Beijing University of Agriculture, Beijing, China; ^2^Beijing Institute of Feed Control, Beijing, China

**Keywords:** high moisture ear corn, moisture content, lactic acid bacteria additives, fermentation quality, bacterial community

## Abstract

Maize (*Zea mays L*) is one of the most widely cultivated crops used as energy feeds. The aim of this study was to evaluate the effects of two lactic acid bacteria additives on the fermentation quality and bacterial community of high moisture ear corn (HMEC) silage at different moisture levels. The study utilized corn kernels and cobs harvested at the stage of complete ripeness as the primary material. The cob was crushed and divided into three treatment groups: an untreated control group (CK), a group treated with a mixture of *Lactobacillus plantarum* and *Lactobacillus brucei* (TQ), or a group treated with a mixture of *Lactococcus lactis* and *Lactobacillus brucei* (KT). Moisture contents were adjusted to 37.5% (L), 42.5% (M) or 47.5% (H) and then silaged for 180 days. Compared to CK, TQ, and KT elevated the dry matter, crude protein, starch, lactic and acetic acid content of HMEC and reduced the pH, neutral detergent fiber, acid detergent fiber and ammonia nitrogen content (*p* < 0.05). Even though both additives improved the bacterial community structure after fermentation, KT experienced the greater enhancement. At a phylum and genus level, KT had the higher relative abundance of *Firmicutes* and *Lactobacillus*, respectively. Compared with the group of 37.5% (L) moisture content, the 42.5% (M) and 47.5% moisture content (H) group increased lactic acid, acetic acid and ammonia nitrogen concentrations and reduced the pH value (*p* < 0.05). In conclusion, the addition of TQ and KT at the appropriate moisture content might be helpful for producing high-quality HMEC. Among the three moisture contents, 42.5% (M) moisture content provides the best silage qualities.

## 1 Introduction

Maize is a widely cultivated crop for silage production due to its high biological yield and high starch concentration. As a result, it has become a primary feed source for ruminants globally (Keshri et al., 2018; Hao et al., 2020; Zhang et al., 2020). In intensive farming, about 60–70% of the energy in livestock and poultry diets is provided by maize, and about 60% of maize energy comes from starch (Qi et al., 2002). However, the starch-protein matrix structure in maize makes it challenging for rumen microorganisms to utilize, ultimately reducing the digestibility of starch in ruminants (Huntington et al., 2006). When large amounts of maize are added to the diet, starch digestibility is generally low, resulting in a waste of feed resources. High moisture corn (HMC) is widely used as it can disrupt this structure and increase digestibility (Ferraretto et al., 2014; Purwin et al., 2022). Various factors affect the production of HMC, including the moisture content of the raw material, fermentation time, and the use of additives. Improper HMC silage can lead to significant resource wastage. As a result, it is crucial to develop appropriate silage methods for HMC.

HMC is prone to oxidative deterioration because of its low moisture content and high starch content when compared to other silages (Kung et al., 2007). Kung et al. (2007) showed that using microbial inoculants could effectively enhance the fermentation quality of HMC. According to Taylor and Kung (2002), *Lactobacillus plantarum* bioinoculants, which produce only lactic acid, could reduce the pH of silage and improve DM recovery. However, the use of homofermentative lactic acid bacteria (LAB) has limited effects on enhancing the aerobic stability of HMC, since lactic acid and low pH are not sufficient to inhibit the growth of yeast (Wardynski et al., 1993). To improve the aerobic stability of HMC, heteromorphic fermentative LAB such as *Lactobacillus brucei* can be used (Taylor and Kung, 2002; Kung et al., 2007). *Lactobacillus brucei* can break down lactic acid into acetic acid with antifungal properties, which improves the stability of silage under anaerobic conditions (Oude Elferink et al., 2001). However, it is not as effective as *Lactobacillus plantarum* in reducing pH during the initial stages of fermentation. This can lead to the growth of harmful bacteria during the aerobic phase of fermentation (Torriani et al., 1992). The growth and reproduction of LAB depend on appropriate moisture levels (Troller and Stinson, 1981). Excessive moisture in raw materials can promote the growth of *Clostridium*, while insufficient moisture can inhibit the growth of LAB (Zheng et al., 2018). Consequently, unsuitable additives and moisture content can have a detrimental effect on HMEC silage.

To enhance high moisture ear corn (HMEC) fermentation, extending the storage period could be beneficial. According to Ferraretto et al. (2014), pH level of HMC decreases with storage periods. Additionally, prolonged silage periods exceeding 120 days contribute to the breakdown of the protein matrix surrounding starch granules, which increases starch digestibility in the rumen (Hoffman et al., 2011). Previous studies have focused on analyzing changes in microbial communities and metabolites in maize silage used for ruminant feed during short-term fermentation periods of less than 60 days (Wu et al., 2020). However, there is a lack of understanding regarding changes in microbial communities and fermentation parameters in medium- and long-term fermented maize silage that is used to supply ruminants throughout the year, highlighting the need for further research in this area.

The objective of this study were to investigate the impacts of different moisture levels and different mixed LAB additives on bacterial communities and fermentation parameters in HMEC after 180 days of storage. This research aimed to contribute to the development of quality control techniques for long-term storage of HMEC.

## 2 Materials and methods

### 2.1 Silage preparation

The material used in the experiment was maize Rip 909 grown harvested in Shanxi Academy of Agricultural Sciences (Temperate continental monsoon climate: 37°82′N, 112°58′E, elevation 372 m, annual mean temperature 4.3–9.2°C, and average annual precipitation 345–588 mm) at complete ripeness stage (initial moisture of 32–35%). Combine corn kernels and cobs were cut into 1–3 cm with a gate cutter, divided into three aliquots, and adjusted with proper amount of ultrapure water. The halogen moisture tester (SF-60 Houwang Electronic Technology, China) was used to accurately and quickly measure the moisture content. Three different moisture contents (L: 37.5%; M: 42.5%; H: 47.5%) were obtained. The inoculant treatments were (1) no additive control (CK); (2) *Lactobacillus plantarum* and *Lactobacillus brucei* at an application rate of 1 × 10^6^ cfu/g of fresh matter each (TQ, silage additive from NDS science and technology development Co., Ltd., China; activity: 1.0 × 10^11^ cfu/g); (3) *Lactococcus lactis* and *Lactobacillus brucei* at an application rate of 1 × 10^6^ cfu/g of fresh matter each (KT, silage additive from Danish Chr. Hansen Holding., Denmark, activity: 1.0 × 10^11^ cfu/g). The ground corn kernels and cobs were then put in fermentation bag with breather valve (20 × 30 cm; Deli Group, China) and sealed using a vacuum sealer (Deli 14,886, Deli Group, China). Three replications were performed for each treatment, and each bag of samples was approximately 1,000 g. In total 27 silage samples (3 replicates × 3 additive treatments × 3 moisture treatments) were made and kept at room temperature (21–25°C).

### 2.2 Chemical composition and fermentation characteristics analysis

After 180 d fermentation, 200 g samples from each group of HMEC were put in an oven and dried at 65°C for 48 h for determination of dry matter (DM). Total nitrogen (TN) and ash contents were determined according to Association of Official Agricultural Chemists [AOAC] (2012). Crude protein (CP) content was calculated by multiplying TN by 6.25. Neutral detergent fiber (NDF), acid detergent fiber (ADF), and acid detergent lignin (ADL) contents were determined with the method of Van Soest et al. (1991). The water-soluble carbohydrate content (WSC) were determined by the phenol-sulfuric acid method of Dubois et al. (1956). Starch content was determined with an enzymatic method (Owens et al., 2002).

20 g HMEC was added to 180 mL of ultrapure water, crushed with juicer for 1 min, and the supernatant was filtered through four layers of gauze and then filtered through qualitative filter paper into a 250 mL sterilized triangular flask (Desta et al., 2016). 100 mL of the filtrate was placed in a 250 mL beaker and the pH was measured with a pH meter (pH-100A, Tianchuang Instruments, China). Another 15 mL of the filtrate was placed in a 25 mL centrifuge tube and stored at −20°C. Lactic acid and NH_3_-N were determined using the method of Kleinschmit and Kung (2006). The volatile fatty acids were determined by gas chromatography (Agilent 6,890 N, Agilent Instruments, United States) with an injection volume of 1 μL and a capillary column (30.00 m × 0.32 mm × 0.25 μm) with a flow rate of 1.5 mL/min and a carrier gas of nitrogen (N_2_) at a column pressure of 81.36 kPa, and the column temperature was started at 150°C, held for 1 min, then increased to 220°C at 6°C/min, held for 15 min, the injection port was in split mode with a split ratio of 5:1.

### 2.3 Microbial community analysis

10 g of HMEC was placed in 40 mL of ultrapure water and shaken at 120 rpm for 120 min. Filtered using three layers of sterile medical gauze and then centrifuged using a low temperature centrifuge 10,000–12,000 r/min at 4°C for 15 min. The DNA extraction was performed using the TIANamp Bacteria DNA Kit (DP302-02, Tiangen, Beijing, China). The PCR was used to amplify the V3–V4 region of the bacterial 16S rRNA gene, with the forward primer 341F (CCTACGGGNGGCWGCAG) and the reverse primer 806R (GGACTACHVGGGTATCTAAT). The PCR products were purified and quantified, and then sequenced at the Shanghai Majorbio Company using Illumina platform (Hiseq2500 PE250). The raw data obtained after Miseq sequencing were spliced by pair-end double-end sequence splicing Flash software (version 1.2.11). According to the overlap relationship the sequences were quality-controlled and screened using Fastp software (version 0.19.6), and the samples were identified based on the barcode and primers at both ends of the sequences, and the sequence orientation was adjusted accordingly. Then valid tags were clustered into operational taxonomic Operational taxonomic units (OTU) based on 97% similarity by Uparse (version 11). Representative sequences of clades and genera were classified by comparison with the Silva database using the plain Bayesian assignment algorithm of RDP Classifier (version 2.13). Based on the operational classification unit (OTU) results, alpha diversity analysis (including Shannon index, Simpson index) was calculated using Mothur (version 1.30.2), and beta diversity including principal coordinate analysis (PCoA) was calculated using Qiime (1.9.1) for beta diversity distance matrix. Sequence data are stored in the NCBI sequence read archive, and the biological project registration number PRJNA989108.

### 2.4 Statistical analysis

The data measured were collated using Excel 2019 software, and statistical analyses were performed using multivariate analysis of variance (ANOVA) of variance with the SPSS program version 20.0 (SPSS Corporation, Chicago, Illinois, United States). The significance of differences of means between groups was tested by Duncan’s test. When *p* < 0.05, the difference was significant.

## 3 Results and discussion

### 3.1 Characteristics of fresh HMEC of before ensiling

The chemical composition of the fresh HMEC before ensiling are shown in Table 1. The contents of several important indicators such as DM, CP, NDF, Starch and WSC in HMEC were 66.63%/FM, 9.54%/DM, 22.87%/DM, 67.73%/DM, and 2.99%/DM, respectively.

**Table 1:** Chemical composition of the fresh HMEC before ensiling.

Items	Fresh HMEC
DM(% FM)^a^	66.63 ± 0.55
CP(% DM)	9.54 ± 0.10
NDF(% DM)	22.87 ± 0.67
ADF(% DM)	13.13 ± 0.25
Ash(% DM)	1.42 ± 0.03
Starch(% DM)	67.73 ± 0.50
WSC(% DM)	2.99 ± 0.08

a FM, fresh matter; DM, dry matter; CP, crude protein; NDF, neutral detergent fiber; ADF, acid detergent fiber; WSC, water-soluble carbohydrate.

### 3.2 Effects of additives and moisture on the nutrient composition of HMEC

The effects of additives and moisture on the chemical composition of HMEC silage are shown in Table 2. When additives were the main effect, the DM, CP and starch contents were significantly higher in the TQ and KT than those in the CK (*p* < 0.05), and the NDF, ADF and WSC contents were significantly lower than those in the CK (*p* < 0.05). When feed moisture content was the main effect, the DM content of each treatment group significantly decreased with increasing moisture content (*p* < 0.05). The CP content of low moisture groups was significantly higher than that in medium moisture and high moisture treatment groups (*p* < 0.05). The WSC content of CK-H was significantly lower than those in CK-M and CK-L (*p* < 0.05). The ADF and starch contents in the TQ-L and KT-L were significantly lower than those in their medium moisture and high moisture treatment groups (*p* < 0.05). The interaction of additives with the moisture content had a significant effect on DM, CP, Starch and WSC contents (*p* < 0.05).

**Table 2:** Effects of different additives and moisture on nutrient contents of HMEC.

Items	Moisture	Treatments^b^			SEM	*P*-value^c^		
		CK	TQ	KT		T	W	T*W
DM (% FM)^d^	L M H	^a^62.90^Ba^ 53.70^Cb^ 50.77^Bc^	64.67^Aa^ 60.53^Ab^ 54.50^Ac^	63.87^Aa^ 58.50^Bb^ 54.60^Ac^	0.324	<0.001	<0.001	<0.001
CP (% DM)	L M H	8.40^Ba^ 7.43^Bb^ 7.23^Bb^	9.53^Aa^ 8.50^Ab^ 8.27^Ab^	9.33^Aa^ 8.37^Ab^ 8.10^Ab^	0.128	<0.001	<0.001	<0.001
NDF (% DM)	L M H	22.17^Aa^ 21.37^Ab^ 21.03^Ab^	21.07^Ba^ 19.70^Bb^ 19.47^Bb^	19.33^Ca^ 17.03^Cb^ 17.40^Cb^	0.291	<0.001	<0.001	0.189
ADF (% DM)	L M H	11.53^A^ 11.93^A^ 12.33^A^	10.37^Bb^ 11.07^Bb^ 11.90^Ba^	8.73^Cc^ 9.10^Cb^ 10.80^Ca^	0.260	<0.001	<0.001	0.114
Ash (% DM)	L M H	1.40 1.36 1.41	1.38 1.39 1.39	1.37 1.40 1.40	0.024	0.943	0.675	0.641
Starch (% DM)	L M H	63.93^B^ 63.63^B^ 62.57^B^	67.97^Aa^ 66.23^Ab^ 65.37^Ab^	67.87^Aa^ 66.50^Ab^ 66.43^Ab^	0.376	<0.001	<0.001	<0.001
WSC (% DM)	L M H	0.74^Aa^ 0.67^Aa^ 0.58^Ab^	0.16^B^ 0.14^B^ 0.11^B^	0.21^Ba^ 0.15^Bb^ 0.17^Bb^	0.017	<0.001	<0.001	0.028

a Values in the same row with different uppercase letter superscripts mean significant difference (*p* < 0.05), and values in the same column under the same item with different lowercase letter superscripts mean significant difference (*p* < 0.05).

b CK, control group; TQ, HMEC with Lactobacillus plantarum and Lactobacillus brucei; KT, HMEC with Lactococcus lactis and Lactobacillus brucei; L, 37.5% moisture contents; M, 42.5% moisture contents; H, 47.5% moisture contents.

c W, effect of corn moisture; T, effect of additives; W*T, effect of the interaction between additives and moisture.

d FM, fresh matter; DM, dry matter; CP, crude protein; NDF, neutral detergent fiber; ADF, acid detergent fiber; WSC, water-soluble carbohydrate.

### 3.3 Effects of additives and moisture on the quality of HMEC fermentation

The effects of additives and moisture on the fermentation quality of HMEC are shown in Table 3. When additives were the main effect, the pH and NH_3_-N of TQ and KT were significantly lower than those of CK (*p* < 0.05), and the lactic acid and acetic acid content were significantly higher than those of CK (*p* < 0.05). When moisture was the main effect, the pH of each group decreased significantly (*p* < 0.05) and NH_3_-N content increased significantly (*p* < 0.05) with increasing moisture content. Acetic acid and NH_3_-N content of KT-L were significantly lower than that of KT-M and KT-H (*p* < 0.05). The lactic acid content of the TQ-M was significantly lower than that of TQ-L and TQ-H (*p* < 0.05). And the lactic acid content of the KT-L was significantly lower than that of the other two moisture treatment groups of KT (*p* < 0.05). The interaction of additives with the moisture content had significant effects on pH, Lactic acid, Acetic acid and NH_3_-N contents (*p* < 0.05).

**Table 3:** Effects of different additives and moisture on fermentation parameters of HMEC.

Items	Moisture	Treatments^b^			SEM	*P*-value^c^		
		CK	TQ	KT		T	W	T*W
pH	L M H	^a^4.42^Aa^ 4.15^Ab^ 4.17^Ab^	4.04^Ba^ 4.09^Aa^ 3.85^Bb^	3.92^Ba^ 3.87^B^ 3.80^Bb^	0.038	<0.001	<0.001	0.003
Lactic acid (% DM)	L M H	1.56^Bb^ 1.68^C^ 1.75^Ca^	2.44^Ac^ 2.22^Bb^ 2.58^Ba^	2.53^Ab^ 2.76^Aa^ 2.78^Aa^	0.034	<0.001	<0.001	<0.001
Acetic acid (% DM)	L M H	0.23^Ca^ 0.14^Cb^ 0.12^Cb^	0.38^Bb^ 0.34^Bb^ 0.45^Ba^	0.44^Ab^ 0.58^Aa^ 0.54^Aa^	0.013	<0.001	0.342	<0.001
Propionic acid (% DM)	L M H	0.04 0.04 0.04	0.05 0.05 0.05	0.05 0.05 0.05	0.005	0.144	0.526	0.852
Butyric acid (% DM)	L M H	ND ND 0.01	ND ND ND	ND ND ND	–	–	–	–
NH_3_-N (% TN)	L M H	13.43^Ac^ 19.97^Ab^ 26.40^Aa^	11.20^Cc^ 17.10^Bb^ 20.60^Ba^	12.07^Bc^ 14.87^Cb^ 17.87^Ca^	0.388	<0.001	<0.001	<0.001

a Values in the same row with different uppercase letter superscripts mean significant difference (*p* < 0.05), and values in the same column under the same item with different lowercase letter superscripts mean significant difference (*p* < 0.05).

b CK, control group; TQ, HMEC with Lactobacillus plantarum and Lactobacillus brucei; KT, HMEC with Lactococcus lactis and Lactobacillus brucei; L, 37.5% moisture contents; M, 42.5% moisture contents; H, 47.5% moisture contents.

c W, effect of corn moisture; T, effect of additives; W*T, effect of the interaction between additives and moisture.

### 3.4 Effects of different treatments on the diversity of bacterial communities in HMEC

**Figure 1:**
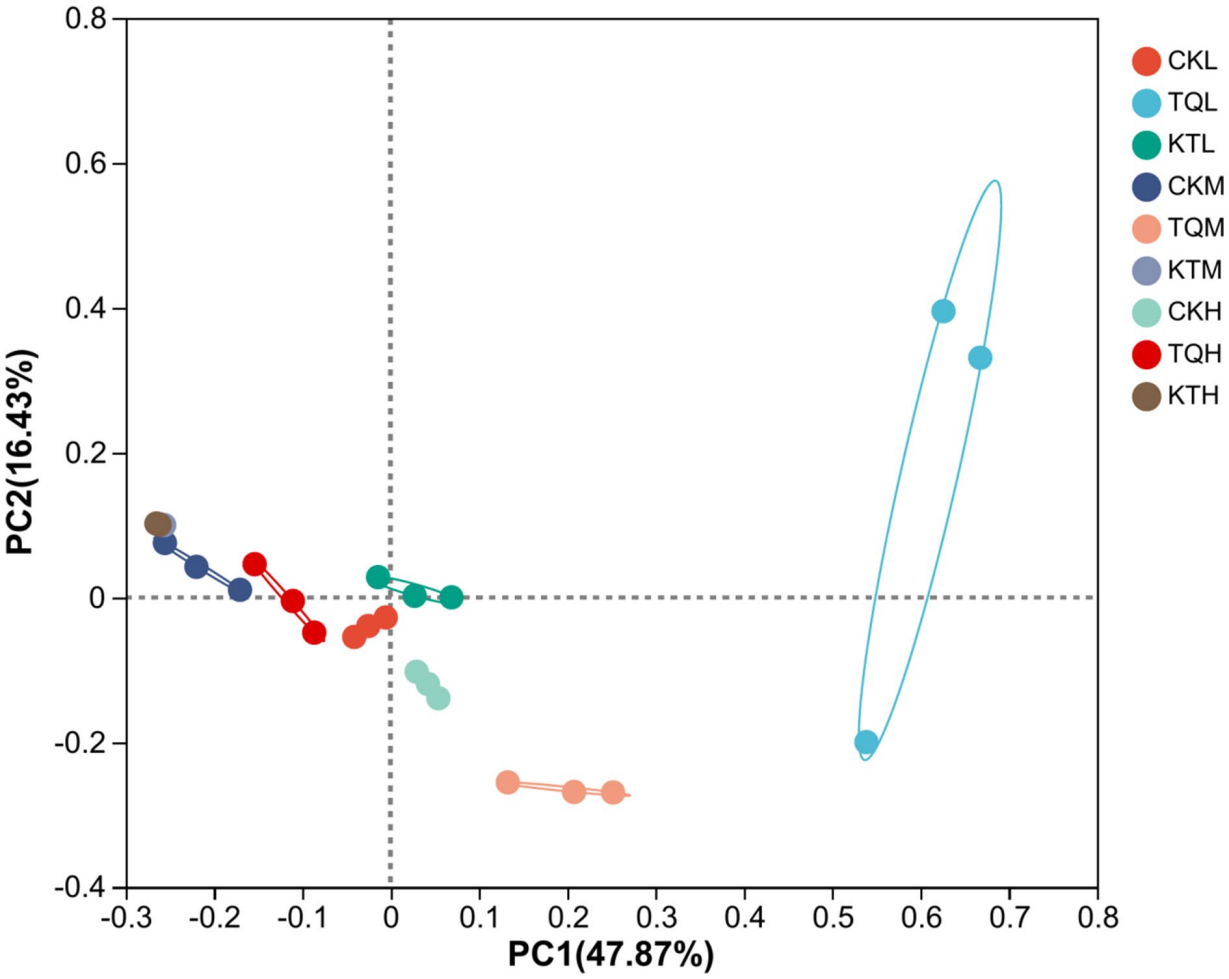
Principal component analysis (PCA) of bacterial community of HMEC under different treatment conditions. CK, control; TQ, *Lactobacillus plantarum* and *Lactobacillus brucei*; KT, *Lactococcus lactis* and *Lactobacillus brucei*; L, 37.5% moisture contents; M, 42.5% moisture contents; H, 47.5% moisture contents.

Table 4 shows the alpha-diversity of bacterial communities in HMEC with different treatments. In total, 1,252,078 valid sequences of 16S rRNA were obtained from 27 samples in this study and clustered into 963 OTU. Shannon and Simpson indices showed that when additives were the main effect, community richness was significantly higher in the TQ group than that in the CK group (*p* < 0.05). Species richness was significantly lower in remaining moisture treatment groups in KT than that those in the CK group, except in the low moisture treatment group (*p* < 0.05); when moisture was the main effect, species richness in the CK-L and KT-L was significantly lower than the remaining two moisture treatments in CK and KT (*p* < 0.05), while species richness in the TQ-H was significantly lower than the remaining two moisture treatments in this group (*p* < 0.05). The interaction of additives with the moisture content significantly effected on Shannon and Simpson index (*p* < 0.05). The coverage of all groups in this experiment was above 99%, indicating that the sequencing results represented the true picture of microorganisms in the samples. The results of *β*-diversity analysis of HMEC for each treatment group are shown in Figure 1, and from the principal component analysis (PCA), it can be seen that PCo1 and PCo2 were 47.87 and 16.43%, respectively. Meanwhile, the bacterial communities of the two additive treatment groups were separated from each other and from the treatment groups without additives, and the bacterial communities of each moisture treatment group were also significantly separated except for the KT-H and KT-M.

**Table 4:** Alpha diversity of bacterial communities in HMEC with different treatments.

Items	Moisture	Treatments^b^			SEM	*P*-value^c^		
		CK	TQ	KT		T	W	T*W
Shannon	L M H	^a^1.43^Bb^ 1.40^Bb^ 1.79^Aa^	2.11^A^ 1.98^A^ 1.70^A^	1.53^Ba^ 0.30^Cb^ 0.19^Bb^	0.172	<0.001	0.005	0.002
Simpson	L M H	0.47^Aa^ 0.38^B^ 0.28^Bb^	0.32^B^ 0.23^Cb^ 0.43^Ba^	0.38^Ab^ 0.88^Aa^ 0.93^Aa^	0.039	<0.001	<0.001	<0.001

a Values in the same row with different uppercase letter superscripts mean significant difference (*p* < 0.05), and values in the same column under the same item with different lowercase letter superscripts mean significant difference (*p* < 0.05).

b CK, control group; TQ, HMEC with Lactobacillus plantarum and Lactobacillus brucei; KT, HMEC with Lactococcus lactis and Lactobacillus brucei; L, 37.5% moisture contents; M, 42.5% moisture contents; H, 47.5% moisture contents.

c W, effect of corn moisture; T, effect of additives; W*T, effect of the interaction between additives and moisture.

### 3.5 Effects of different treatments on the relative abundance of bacteria in HMEC

**Figure 2:**
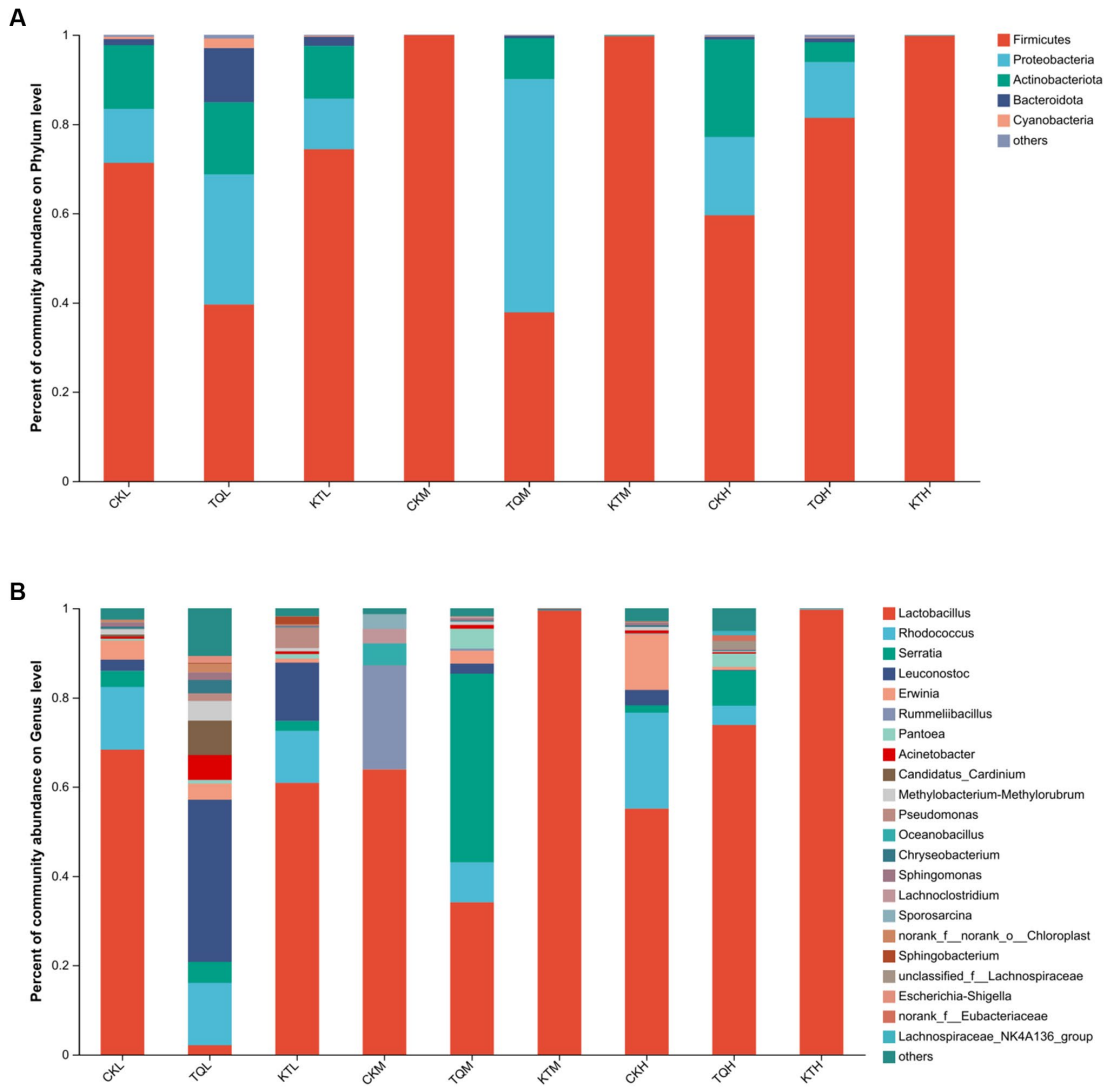
Relative abundance of bacterial community by phylum **(A)** and genus **(B)** for HMEC. CK, control; TQ, *Lactobacillus plantarum* and *Lactobacillus brucei*; KT, *Lactococcus lactis* and *Lactobacillus brucei*; L, 37.5% moisture contents; M, 42.5% moisture contents; H, 47.5% moisture contents.

The relative abundance of bacterial community of HMEC with different treatments is shown in Figure 2. From the community bar diagram, it can be seen that after 180 days of silage, *Phylum Firmicutes*, *Proteobacteria*, and *Actinomycetes* were the top three bacterial phyla attached to HMEC in each treatment group. The relative abundance of the *Phylum Firmicutes* increased significantly with increasing moisture content in all treatment groups, except for the TQ-M and CK-H treatment groups (Figure 2A). The relative abundance of the *Phylum Firmicutes* was high in CK-H, KT-M, and KT-H, all exceeding 90%, and lowest in the TQ-M group (37.79%). The relative abundance of *Proteobacteria* was highest in TQ-M (52.30%). The relative abundance of *Proteobacteria* in TQ group was significantly higher than that in KT and CK groups, while the relative abundance of *Phylum Firmicutes* was significantly lower than that in KT group. Figure 2B shows the abundance of bacterial communities at different genus levels. *Lactobacillus* (62.28%), *Rhodococcus* (14.06%), and *Erwinia* (4.29%) were the top three dominant genera in CK-L; *Lactobacillus* (63.85%), *Rummeliibacillus* (23.32%), and *Oceanobacillus* (4.9%) were the top three dominant genera in CK-M; *Lactobacillus* (55.10%), *Rhodococcus* (21.52%), and *Erwinia* (12.5%) were the top three dominant genera of CK-H. When the additives are added, the relative abundance of *Lactobacillus* in KT increased comparing with CK. The relative abundance of HMEC *Lactobacillus* for the three moisture species ranked as KT-H > KT-M > KT-L. In TQ the relative abundance of *Lactobacillus* in the partial moisture treatment group was reduced relative to CK. The relative abundance of TQ-L (3.01%) and TQ-M (34.1%) *Lactobacillus* was decreased compared to both CK-L (68.3%) and CK-M (63.9%). The relative abundance of TQ-H *Lactobacillus* (73.82%) was higher than that of CK-H (55.1%). The dominant genus for TQ-L changed to *Leuconostoc* (53.81%) and for TQ-M to *Serratia* (42.27%).

**Figure 3:**
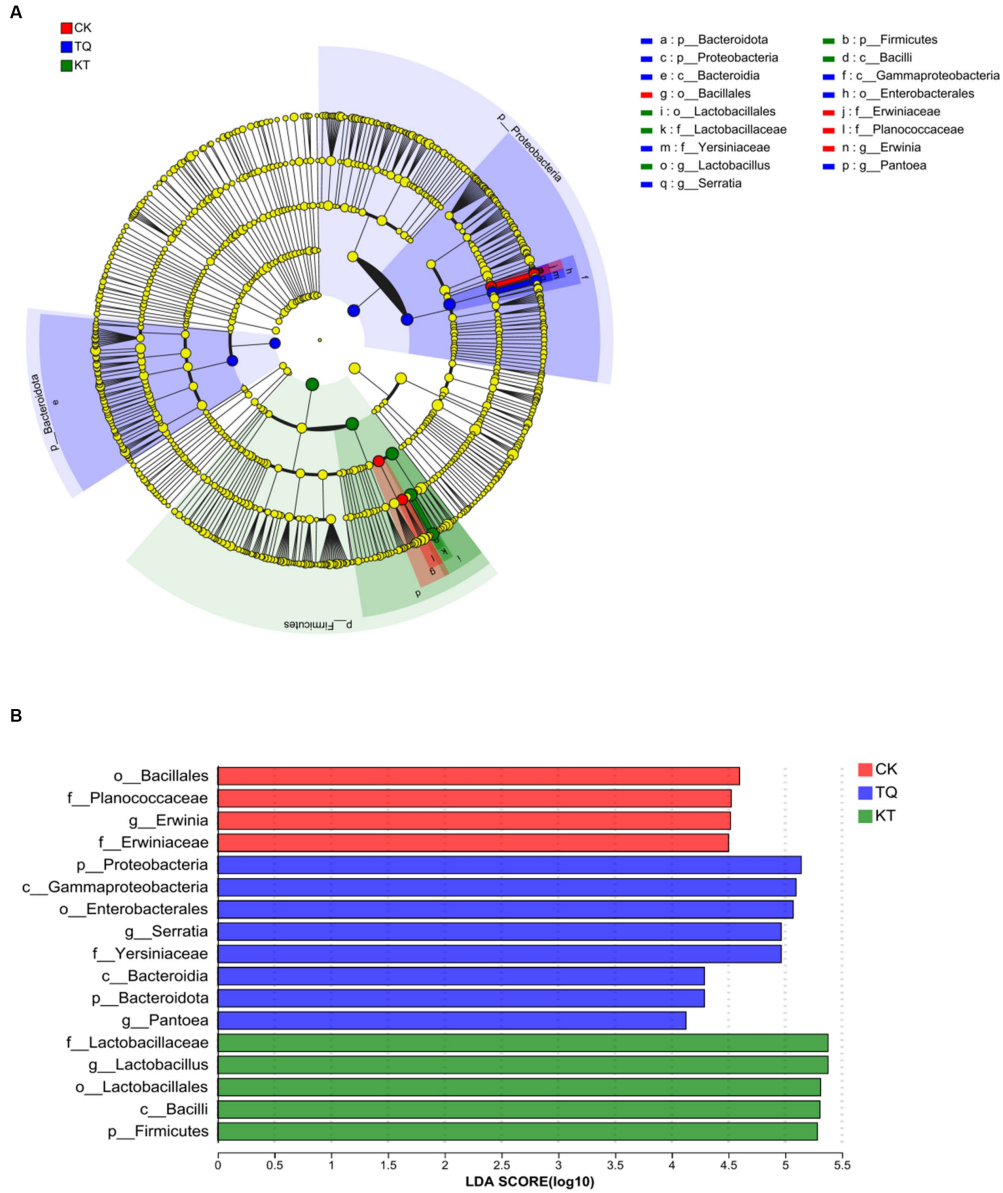
Distribution and evolutionary branching of LDA values of different species in HMEC treated with different additives. **(A)** Diagram of the evolutionary branching of different species. The dots radiating from the inner to outer circles represent the level of classification from phylum to genus. Each dot on the different taxonomic levels represents a group at each level, and the diameter of the circle reflects the relative abundance of the group. The species with insignificant differences were yellow dots. Red dots represent microbial groups that play an important role in the CK (control), blue dots represent microbial groups that play an important role in the TQ (*Lactobacillus plantarum* and *Lactobacillus brucei*), and green dots represent microbial groups that play an important role in the KT (*Lactococcus lactis* and *Lactobacillus brucei*). **(B)** Distribution of LDA values of different species. The LDA discriminant histogram counts the microbial taxa with significant effects in each group, and the longer the length of the histogram, the greater the effect of species abundance on the differential effect.

The LEfSe method was used to assess differences in microbial communities between TQ and KT (Figure 3; LDA threshold of 4). In the CK group, four bacterial species were significantly enriched, with *Bacillales* (LDA score, 4.60) having the highest LDA score. In the TQ group, eight bacterial species were significantly enriched, with the highest LDA score for *Probeobacteria* (LDA score, 5.14). In the KT group, four bacterial species were significantly enriched, with *Lactobacillus* (LDA score, 5.38) having the highest LDA score. These results suggest that HMEC silage supplemented with different additives differed somewhat in the abundance of species in specific communities.

### 3.6 Association between microbial communities and chemical indicators

**Figure 4:**
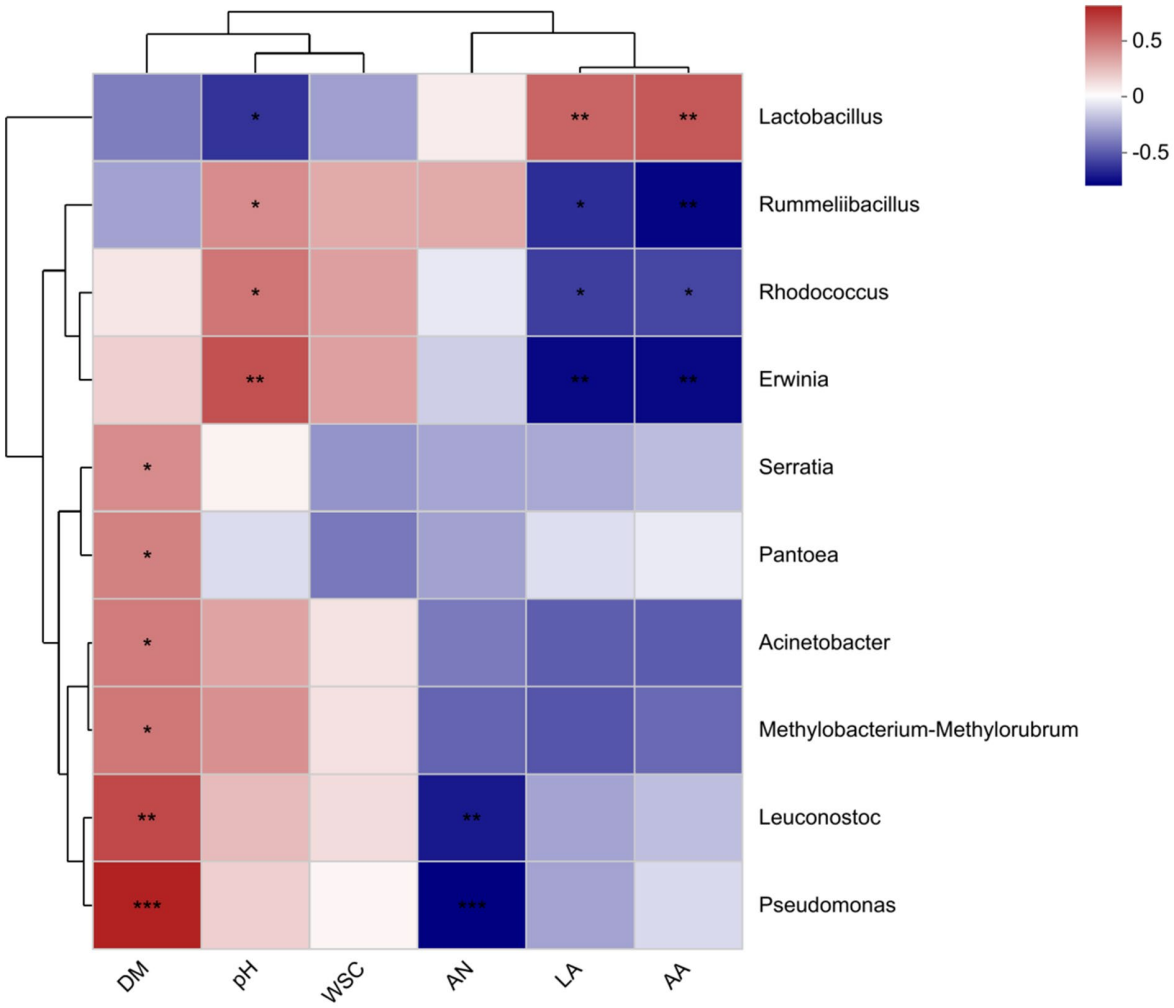
Spearman correlation heat map of bacterial genus abundance and chemical indicators. DM, dry matter; WSC, water-soluble carbohydrate; AN, ammonia nitrogen; LA, lactic acid; AA, acetic acid.

Heat maps synthesized based on Spearman correlations (genus level; Figure 4) show the associations between the top 10 species and fermentation parameters at the total abundance of microbial community taxonomic level. In this study, *Lactobacillus* was negatively correlated with pH (*R* = −0.476), and positively correlated with lactic acid (*R* = 0.521) and acetic acid (*R* = 0.559). The *Leuconostoc* was positively correlated with DM content (*R* = 0.603) and negatively correlated with NH_3_-N content (*R* = −0.544). The DM content was positively correlated with *Acinetobacter* (*R* = 0.45) and *Serratia* (*R* = 0.403).

## 4 Discussion

### 4.1 Effects of different treatments on the nutrient composition of HMEC

DM is essential for the efficient use of fermented feeds. Research demonstrated that a higher DM content could effectively hinder the growth and reproduction of harmful bacteria such as *Clostridium* and *Escherichia coli*, as well as reduce the breakdown of nutrients by these bacteria (Cai and Kumai, 1994; Yitbarek and Tamir, 2014). The present experiment found that the DM content in the TQ and KT groups, which were treated with different additives, was significantly higher than DM in the CK. This is due to the use of mixed LAB additives, which allow beneficial bacteria such as LAB to grow and multiply rapidly and produce large amounts of lactic acid, inhibiting the growth and multiplication of harmful bacteria, and reducing DM consumption.

In silage, good fermentation quality is always accompanied by good nutrient preservation. In this study, it was found that the addition of LAB could improve the CP content of HMEC to some extent. It is speculated that LAB may have improved the nutritional value of HMEC by reducing the degradation of protein during silage or inhibiting the decomposition of spoilage microorganisms, thus reducing the NH_3_-N content of total nitrogen and indirectly increasing the CP content. This is consistent with the study of Li et al. (2012), who found that after 150 d of fermentation in maize inoculated with a complex LAB preparation, the NH_3_-N content was significantly lower and the CP content was significantly higher compared to the control.

The lower NDF and ADF content in the TQ and KT compared to the CK may be due to their higher organic acid production, which hydrolyzes more structural carbohydrates (Jiang et al., 2020). In general, the acid hydrolysis of structural carbohydrates is accompanied by the release of WSC (Desta et al., 2016). However, in this experiment, the WSC content in the TQ and KT was lower than that in the CK, which was due to the fact that a large amount of WSC was converted to organic acid, ethanol and carbon dioxide by LAB after the addition of LAB additives, which led to a decrease in the WSC content (Muck et al., 2018; Wang S. et al., 2018). This is in agreement with the findings of Kleinschmit and Kung (2006).

### 4.2 Effects of different treatments on HMEC fermentation parameters

The pH, lactic acid, and NH_3_-N are the most important indicators of silage fermentation quality (Hashemzadeh-Cigari et al., 2014). A suitable pH not only inhibits the growth of undesirable microorganisms, but also reduces the content of volatile fatty acids in the silage, keeps the organic acid content in the appropriate fermentation range, and reduces the decomposition of nutrients in the forage, resulting in better preservation of the forage. In this experiment, except for the medium moisture treatment group TQ, the pH of all the moisture treatment groups was significantly lower than the CK, and the lactic acid content was significantly higher than the CK. Homofermentative LAB can ferment WSC to lactic acid rapidly in the early silage stage, so that the pH drops rapidly, creating conditions for the subsequent initiation of *Lactobacillus brucei* (Torriani et al., 1992; Borreani et al., 2018). Therefore, the use of mixed fermentative LAB increases the lactic acid content of HMEC during the fermentation process. The increase in pH and the decrease in lactic acid content in TQ-H may due to *Leucococcus* as the dominant genus in the bacterial community. Cai et al. (1998) found that usually *Leucococcus* was not as capable of acid production as *Lactobacillus* and did not cause as significant a pH decrease as *Lactobacillus*. Subsequently, with the increase of moisture, the content of LAB in the medium moisture treatment group of TQ was significantly higher due to the more active growth of LAB at higher moisture content (Ferraretto et al., 2018), but at this time, the number of *Leucococcus* decreased substantially with the increase of moisture, due to the good antibacterial effect of the *Leucococcus*, it can inhibit the growth and reproduction of *Escherichia coli* such as *Serratia*, and the reduction of its number causes *Escherichia coli* such as *Serratia* to compete with LAB and *Leucococcus* for nutrients,thus increasing the number of *Serratia* and inhibiting the growth of *Lactobacillus* and *Leucococcus* (Heron et al., 2010; Liu et al., 2022). This resulted in an increase in pH and the lactic acid content decreased in the moisture treatment group in TQ.

Acetic acid is a promoter of aerobic stability during fermentation and an effective inhibitor of fungi (Le Lay et al., 2016). The addition of additives in this experiment significantly increased the content of acetic acid because both additives contain *Lactobacillus brucei*, which is a heterotypic fermenting LAB, and its metabolites include lactic acid and acetic acid. The acetic acid content of the KT group was higher than that of the TQ group due to the rapid growth and reproduction of *Lactococcus lactis* in the KT at the early stage of fermentation, which makes the pH drop rapidly and inhibits other microorganisms reproduction (Zhao et al., 2021) and allowed *Lactobacillus brucei* to start earlier. The decrease in NH_3_-N content in the treated group could be attributed to the inoculation of the LAB additive that inhibited the growth of *Clostridium* and the trypsin activity of *Mycobacterium* (Oliveira et al., 2017).

### 4.3 Effects of different treatments on the diversity of bacterial communities in HMEC

The fermentation process encompasses a complex symbiotic system involving numerous microorganisms. Microbial structural composition and diversity affect fermentation quality and nutrient composition. It is generally considered that the microbial diversity of raw materials should be drastically reduced after silage and replaced by LAB (Jiang et al., 2020). In this experiment, the species diversity of the KT treatment group was significantly lower than that of the CK and TQ groups as shown by the Shannon and Simpson indices, which might be because the additives in the KT treatment group contained *Lactococcus lactis*, and the acid production rate of *Lactococcus lactis* was greater than that of *Lactobacillus plantarum* at the early stage of fermentation under low oxygen conditions. *Lactococcus lactis* could make the initial pH of silage drop rapidly. With the rapid formation of the acidic anaerobic state, *Lactococcus lactis* became the dominant flora and further acid production lowered the pH to below 4.2, thus inhibiting the reproduction of spoilage microorganisms in the aerobic stage and reducing the bacterial diversity in the silage (Zhao et al., 2021).

The results of PCA can be used to distinguish bacterial communities in different treatment groups. HMEC from each treatment in this experiment were individually aggregated within a narrow range, indicating good reproducibility of the samples and significant separation and differences in the distribution and composition of the microbial community across treatments. This result suggested that different total classes of additives as well as different moisture could significantly affect the microbial composition of HMEC, which is in agreement with the results of Xu et al. (2022) about the effect of moisture and additives on the microbial composition of the community in silage.

### 4.4 Effects of different treatments on the relative abundance of bacteria in HMEC

Silage is formed by microbial communities in an extremely complex environment, and bacteria play an important role in the whole fermentation process (Yi et al., 2019). It has been shown that the *Phylum Firmicutes* can produce acid and secrete a variety of enzymes in an anaerobic environment, and that anaerobic and low pH environments help to promote the growth and reproduction of thick-walled bacteria (Wang Y. et al., 2018; Ali et al., 2020). In the present experiment, the relative abundance of *Phylum Firmicutes* was also significantly higher with the increase of moisture in the KT group, possibly due to the rapid fermentation of *Lactococcus lactis* which lowered the pH of HMEC and provided strong conditions for the growth and propagation of *Phylum Firmicutes* (Lin et al., 1992; Cai and Kumai, 1994). The *Phylum Firmicutes* gate can break down macromolecules such as cellulose and starch (Romero et al., 2017). Therefore, it is inferred that the changes in cellulose composition in the KT-treated group in this experiment were related to the high abundance of the *Phylum Firmicutes*. The decrease in abundance of the *Phylum Firmicutes* and the increase in abundance of the *Anamorphic phylum* in the water-treated group in TQ may be due to the lack of tolerance to low pH in the strains of the TQ additive, leading to the growth of other harmful bacteria.

In addition, we investigated the variation of bacterial communities in HMEC at the genus level. *Lactobacilli* are known to play a key role in increasing lactic acid and lowering pH, and high abundance of *Lactobacillus* has become a symbol of high quality silage (Mu et al., 2020). In the present study, we found that the relative abundance of *Lactobacillus* increased significantly with increasing moisture in the TQ and KT treatment groups. This may be due to the fact that with increasing moisture, the metabolism of *Lactobacillus* accelerated, rapidly lowering the pH of the silage, preventing the growth of undesirable microorganisms and creating an ideal environment for *Lactobacillus* to flourish. However, there was a significant decrease in the abundance of *Leucococcus* and a significant increase in the abundance of *Serratia* in the in TQ-H. It is inferred that this may be because the moisture content of the TQ-L group inhibited the growth of *Lactobacillus* and made *Leucococcus* the dominant genus. Subsequently, with the increase of moisture, the growth of *Leucococcus* was inhibited and the pH increased slightly, and the inhibition of *Escherichia coli* such as *Serratia marcescens* was weakened, and it competed with *Lactobacillus* and *Leucococcus* marcescens for nutrients, resulting in *Escherichia coli* such as *Serratia marcescens* becoming the dominant genus (Heron et al., 2010; Weyker et al., 2016). Hou et al. (2022) studied the changes in microbial communities during silage fermentation in native forages on the Inner Mongolian plateau, and their results also indicated that the abundance of *Leucococcus* significantly decreased with the increase in forage moisture.

### 4.5 Relationship between fermentation parameters and bacterial communities

Studies have shown an interaction between bacterial communities and fermentation parameters (McAllister et al., 2018). Spearman correlation heat map in this experiment showed the correlation of pH, LA, AA, NH_3_-N, and WSC with bacterial community. The abundance of *Lactobacillus* was positively correlated with lactic acid content and acetic acid content and negatively correlated with pH, which is consistent with the findings of Mu et al. (2020). This is mainly due to the fact that lactic acid produced by the metabolism of LAB can rapidly lower the pH of HMEC and that rapid acidification plays a key role in inhibiting the biological activity of harmful bacteria and preserving feed nutrients (Lv et al., 2020). The negative correlation between acetic acid content and abundance of harmful bacteria such as *Pseudomonas aeruginosa* may be due to the inhibitory effect of acetic acid on harmful bacteria such as *Pseudomonas aeruginosa*. A similar finding was made by Feng et al. (2022) who showed that acetic acid could inhibit the growth of *Pseudomonas aeruginosa* by altering its membrane permeability, membrane potential and reduction potential. A positive correlation between DM content and abundance of *pseudomonas* is also consistent with our results showing that the higher water content inhibited the growth of *pseudomonas*. The negative correlation between DM abundance and NH_3_-N content may be due to the good antibacterial effect of DM, which inhibits the hydrolysis of proteins by harmful bacteria such as *E. coli* (Liu et al., 2022).

## 5 Conclusion

Combining the nutrient content, silage quality and bacterial diversity of HMEC, it can be concluded that both water content and additives had significant effects on the silage quality of HMEC. Both TQ and KT additives enhanced the fermentation quality of HMEC and increased the abundance of *Lactobacillus* TQ additive was more suitable for high moisture HMEC. The number of LAB increased with higher moisture for both additives. When the raw material moisture was 42.5% and above, the abundance of *Lactobacillus* exceeded 90% in the KT group. Considering that the higher the moisture was, the more serious the hydrolysis of protein and other nutrients could be, the moisture could be limited at about 42.5% with KT additive, which could better preserve the nutrients of HMEC.

## Data availability statement

The datasets presented in this study can be found in online repositories. The names of the repository/repositories and accession number(s) can be found in the article/Supplementary material.

## Author contributions

JL and KG designed the trial protocol and completed the article. JL, ZL, SS, and XZ performed the experiments. JL, WZ, XrZ, JB, and ZY completed the collation and statistical analysis of the trial data. KG completed the writing and grammatical corrections to the article. All authors contributed to the article and approved the submitted version.
